# Algorithm-driven management of pediatric multiple magnetic foreign bodies: a single-center experience of 171 cases

**DOI:** 10.1186/s12887-026-07268-6

**Published:** 2026-07-08

**Authors:** Mengjun Dong, Kairui Yang, Hengpan Yao, Yijing Liu, Di Liu, Fang Zhou

**Affiliations:** Department of Digestion, Children’s Hospital Affiliated to Zhengzhou University, Henan Children’s Hospital Zhengzhou Children’s Hospital, Zhengzhou, Henan 450018 China

**Keywords:** Children, Gastrointestinal Foreign Body, Abdominal pain, Perforation

## Abstract

**Objective:**

To delineate the clinical features of pediatric multiple magnetic foreign body ingestion and evaluate the clinical decision-making criteria for endoscopic, surgical, or conservative management.

**Methods:**

A retrospective cohort study was conducted on pediatric patients diagnosed with multiple magnetic foreign body ingestion at the Children’s Hospital Affiliated to Zhengzhou University between January 2019 and October 2025. Following the collection of comprehensive clinical data, the cohort was stratified into subgroups based on the specific intervention methods employed for foreign body removal. Demographic data, clinical manifestations, triage modalities, and outcomes were analyzed.

**Results:**

The cohort comprised 171 patients (120 males; median age 3.42 years), with a median ingestion-to-intervention interval of 1.5 days. A definitive ingestion history was lacking in 41.52% of cases. The cohort was triaged into a non-surgical group (*n* = 50; 40 endoscopic retrievals and 10 spontaneous passages under conservative observation) and a surgical group (*n* = 121). Gastrointestinal perforations occurred in 108 cases (63.16% of this referred cohort), predominantly in the small intestine. Symptomatic presentation (abdominal pain, vomiting) and absent bowel sounds were strong clinical indicators for severe transmural injury and surgical intervention. All patients were successfully discharged without mortality, although 12 cases required reoperation for postoperative adhesive intestinal obstruction.

**Conclusion:**

Multiple magnetic foreign body ingestion carries a profound risk of occult transmural perforation, demanding aggressive, algorithm-driven triage in tertiary care settings. Urgent endoscopic retrieval is prioritized for accessible proximal magnetic foreign bodies, while asymptomatic distal ingestions may be managed with strict inpatient conservative observation. However, the onset of abdominal symptoms, failure of radiographic progression, or unsuccessful endoscopic retrieval mandates immediate surgical exploration. During surgery, a complete intraoperative bowel exploration is imperative to prevent missed multifocal injuries.

**Supplementary Information:**

The online version contains supplementary material available at https://doi.org/10.1186/s12887-026-07268-6.

## Introduction

With economic development and rising living standards, magnetic toys have become increasingly popular among parents and children. However, the emergence of neodymium iron boron (NdFeB) magnets in children’s toys has led to a rising incidence of magnet ingestion incidents [1, 2]. According to a survey from the National Electronic Injury Surveillance System, there were more than 23,756 cases of magnet ingestion in the United States from 2009 to 2019, with an annual increase of 6.1% [3]. It is noteworthy that approximately one-quarter of these cases involved the ingestion of multiple magnets or magnets combined with other metallic objects.

Repeated ingestion of magnetic foreign bodies (either multiple magnets or magnets combined with other metallic objects) carries a higher risk of mutual attraction compared to a single ingestion. This attraction can compress the vascular supply of intestinal loops, leading to ischemia, tissue necrosis, perforation, fistula formation, intestinal obstruction, intestinal volvulus, secondary peritonitis, and even death [4–7]. Our single-center data from 2019 to 2025 showed no marked reduction in pediatric magnetic foreign body ingestions (see Supplementary Fig. 1). This highlights the persistent challenge in early diagnosis and optimal treatment decision-making for clinicians [8]. Given the slow progression and insidious nature of magnetic foreign body ingestion within the gastrointestinal tract, early diagnosis, selection of appropriate treatment modalities, and determination of optimal surgical timing are critical.

To our knowledge, this study represents one of the larger clinical cohorts focused specifically on pediatric multiple gastrointestinal magnetic foreign body ingestion to date. This substantial sample size enhances the statistical reliability of our conclusions and provides a robust representation of this clinical challenge. Additionally, while extant literature predominantly focuses on surgical outcomes, we offer a more nuanced analysis by stratifying patients into non-surgical and surgical groups. This classification facilitates a precise understanding of various management patterns and provides critical insights into indicators necessitating surgery following non-surgical failure. Furthermore, our findings provide empirical evidence for the “inpatient conservative observation” strategy advocated in recent international guidelines. By documenting outcomes of asymptomatic cases monitored via serial radiographs every 6 h, we contribute valuable data toward defining the safe observation window for distal magnets. Finally, neodymium magnets—renowned for superior attractive force and high morbidity—accounted for 84.21% of our cohort. By objectively documenting severe complication rates, this study further elucidates the extreme risks of these objects and underscores that “prevention is more critical than treatment.”

## Methods

### Ethics statement

This study protocol was designed in strict adherence to the ethical principles stipulated in the Declaration of Helsinki and was reviewed and approved by the Medical Ethics Committee of the Children’s Hospital Affiliated to Zhengzhou University, with the ethical approval number 2019-K-033.

### Patients and data collection

A retrospective analysis was conducted on children aged 0–18 years who were hospitalized and diagnosed with multiple magnetic foreign bodies at the Children’s Hospital Affiliated to Zhengzhou University from January 2019 to October 2025. Multiple magnetic foreign bodies were defined as ingestion of two or more magnetic foreign bodies, or a combination of magnetic and metallic foreign bodies.

#### Inclusion criteria

1. Abdominal X-ray and/or computed tomography (CT) showing multiple gastrointestinal magnetic foreign bodies;
2. Diagnosis of multiple magnetic foreign bodies or mixed magnetic and metallic foreign bodies confirmed by gastroscopy, surgical exploration, or post-spontaneous passage verification.

#### Exclusion criteria

Patients with incomplete clinical data or who self-discharged against medical advice were excluded.

#### Grouped by treatment modality

1. Surgical group: Patients who underwent laparoscopic or open surgical foreign body removal;
2. Non-surgical group: Patients who had foreign bodies removed by gastroscopy or spontaneously passed foreign bodies after conservative treatment.

### Data collection

Clinical data of the patients were collected, including age, gender, clinical symptoms and signs, type and quantity of foreign bodies, perforation site, treatment modality, and prognosis. The type, size, quantity, and potential location of foreign bodies were determined by thoracoabdominal X-ray with or without CT.

### Selection and implementation of treatment regimens for pediatric multiple magnetic foreign bodies

**Fig. 1:**
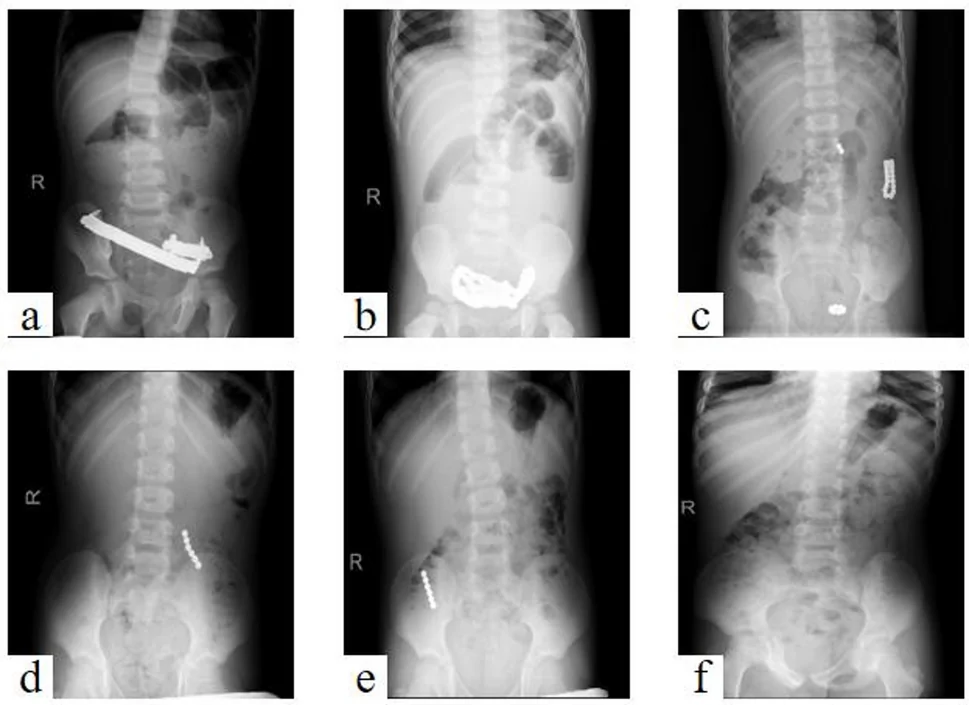
Upright abdominal X-ray findings in pediatric patients with multiple magnetic foreign bodies in the digestive tract. **a** Irregular high-density shadow in the lower abdomen (such as foreign bodies including magnetic beads, coins, and button batteries); **b**, Aggregation of multiple magnetic foreign bodies in the lower abdomen; **c**, Distribution of multiple magnetic foreign bodies in the abdomen due to repeated ingestions; **d**–**f**, Dynamic imaging monitoring of a child with multiple magnetic foreign bodies in the digestive tract

Clinical management of multiple magnetic foreign body ingestion is tailored to patient history, clinical symptoms, physical signs, and imaging findings, with all invasive interventions performed under general anesthesia. For asymptomatic patients whose imaging confirms that magnets have passed distal to the stomach, inpatient conservative observation is implemented. Serial thoracoabdominal plain radiography is performed every 6 h to track foreign body progression, which is considered the clinical ‘gold standard’ for monitoring transit (Fig. 1) [4, 5, 9]. This approach may be supplemented by osmotic laxatives, such as polyethylene glycol, to facilitate passage. Observation must be escalated to surgical intervention if serial imaging demonstrates a failure of magnet progression or if the patient develops clinical indicators of obstruction or perforation, including abdominal pain, vomiting, tenderness, or peritonitis.

For magnets within endoscopic reach (esophagus, stomach, and duodenum), urgent endoscopic removal should be performed, ideally within 12 h, to prevent distal migration and subsequent injury. Electronic gastroscopes (PENTAX EG-2490 K or EG27-i10) are utilized, with auxiliary instruments—including foreign body baskets, transparent caps, and snare loops—selected based on the size, shape, and impaction site of the magnets. Following retrieval, the mucosa must be thoroughly re-inspected for damage, active bleeding, or residual fragments. If an endoscopic perforation is identified, this may involve endoscopic closure combined with conservative measures—such as nasogastric decompression, enteral nutrition via nasojejunal tube, and antibiotic/acid-suppressant therapy—or immediate conversion to surgery. Surgical intervention is indicated for failed endoscopic retrieval, failure of progression, or definitive signs of gastrointestinal obstruction or perforation. Intraoperative procedures, including primary repair, intestinal resection and anastomosis, or temporary ostomy, are performed based on the severity of mucosal injury and contamination, followed by postoperative decompression, anti-infective therapy, and parenteral nutrition.

### Statistical analysis

Statistical analysis was performed using SPSS 27.0 software. The statistical methods selected were based on the characteristics of the data: t-tests or Mann–Whitney U tests were used for continuous data depending on normality. Intergroup comparisons for categorical data were performed using chi-squared tests. A multivariable logistic regression model was constructed to identify independent predictors for surgical intervention. Variables demonstrating statistical significance in the univariate analysis were considered as candidates. However, to prevent model overfitting in strict accordance with the events-per-variable (EPV) rule and to avoid multicollinearity among overlapping clinical signs, only variables demonstrating statistical significance and clinical representativeness were incorporated into the final model. False discovery rate (FDR)-adjusted P-values were used to assess significance, and a two-tailed *P* < 0.05 was considered statistically significant.

## Results

### Patient demographics and clinical manifestations

Between January 2019 and October 2025, a total of 171 pediatric patients (120 males and 51 females) were treated for multiple magnetic foreign body ingestion (five patients were excluded due to discharge against medical advice). The median age was 3.42 years (IQR: 2.00–6.00 years). A definitive history of ingestion was present in only 100 cases (58.48%), while 71 cases (41.52%) lacked a clear witnessed event. Upon admission, abdominal pain (47.95%) and vomiting (39.77%) were the predominant symptoms, often accompanied by tenderness (44.44%) and decreased or absent bowel sounds (42.69%).

### Grouping and clinical manifestations

Based on the clinical algorithm, patients were stratified into a non-surgical group (*n* = 50) and a surgical group (*n* = 121). Regarding endoscopic interventions, all cases selected for initial endoscopic management involved proximal (upper gastrointestinal) foreign bodies. Within the non-surgical group (*n* = 50), all patients underwent initial gastroscopy: 40 cases achieved successful endoscopic retrieval, while in the remaining 10 cases, the magnets had already migrated beyond endoscopic reach and were subsequently passed spontaneously under dynamic radiographic surveillance. Conversely, within the surgical group (*n* = 121), 28 patients initially underwent gastroscopy but mandated conversion to surgical exploration due to irretrievable or only partially retrievable magnets, frequently accompanied by suspected perforation, the onset of clinical symptoms, or subsequent radiographic stagnation. Detailed demographic and clinical comparisons between the two groups are summarized in Table 1. Patients requiring surgery presented with significantly higher rates of vomiting, abdominal pain, and signs of peritonitis (*P* < 0.05).

**Table 1:** Comparison of clinical characteristics between the non-surgical and surgical groups in pediatric patients following multiple magnetic foreign body ingestion

	Non-surgical Group (n=50)	Surgical Group (n=121)	Statistic	*P* _ Raw	*P* _ FDR
Male [n, (%)]	36(72.00%)	84(69.42%)	χ²=0.112	*P*=0.737	*P*=0.737
Age at presentation, years [Median (Q1, Q3)]	4.29(2.42, 6.09)	3.08(2.17,4.83)	Z=-2.300	*P*=0.021	*P*=0.026
History of foreign body ingestion^a^	49(98.00%)	51(42.15%)	χ²=45.453	*P*<0.001	*P*<0.001
Time to foreign body ingestion^b^, Days [Median (Q1, Q3)]	0.17(0.08, 0.29)	4.00(2.00, 7.00)	Z=-6.695	*P*<0.001	*P*<0.001
Fever [n, (%)]	3 (6.00%)	18(14.88%)	χ²=2.587	*P*=0.108	*P*=0.126
Vomiting [n, (%)]	2 (4.00%)	64(52.89%)	χ²=35.686	*P*<0.001	*P*<0.001
Abdominal pain [n, (%)]	6 (12.00%)	76(62.81%)	χ²=36.597	*P*<0.001	*P*<0.001
Lethargy [n, (%)]	0(0.00%)	20(16.53%)	χ²=9.359	*P*=0.002	*P*=0.003
Tenderness [n, (%)]	5(10.00%)	71(58.68%)	χ²=33.953	*P*<0.001	*P*<0.001
Abdominal rigidity [n, (%)]	1(2.00%)	42(34.71%)	χ²=20.112	*P*<0.001	*P*<0.001
Abdominal distension [n, (%)]	1(2.00%)	38(31.40%)	χ²=17.376	*P*<0.001	*P*<0.001
Diminished or Absent bowel sounds [n, (%)]	3(6.00%)	70(57.85%)	χ²=38.880	*P*<0.001	*P*<0.001
Hyperactive bowel sounds [n, (%)]	0(0.00%)	3(2.48%)	χ²=1.262	*P*=0.261	*P*=0.288
Leukocytosis [n, (%)]	12(24.00%)	58(47.93%)	χ²=8.382	*P*=0.004	*P*=0.006
Elevated CRP [n, (%)]	0(0.00%)	21(17.36%)	χ²=9.893	*P*=0.002	*P*=0.003
Hemoglobin reduction [n, (%)]	13(26.00%)	26(21.49%)	χ²=0.409	*P*=0.522	*P*=0.548
Number of foreign bodies	4.00(3.00, 8.00)	7.00(4.00, 13.00)	Z=-3.971	*P*<0.001	*P*<0.001
Length of hospital stay, Days	3.00(2.00,4.00)	10.00(9.00,11.00)	Z=-8.341	*P*<0.001	*P*<0.001
Presence of Perforation [n, (%)]	6(12.00%)	102(84.30%)	χ²=79.476	*P*<0.001	*P*<0.001
Number of Perforations	0.00(0.00,0.00)	2.00(1.00, 4.00)	Z=-8.238	*P*<0.001	*P*<0.001

^a^ A total of 100 cases (58.48%) had a definitive history of foreign body ingestion, while 71 cases (41.52%) lacked a clear history

^b^ Among patients with a clear history of foreign body ingestion time, n=48 in the Non-surgical Group and n=51 in the Surgical Group

Continuous variables are presented as median (interquartile range), and intergroup comparisons were performed using the Mann-Whitney U test (statistic Z). Categorical variables are presented as [n, (%)], with intergroup comparisons performed using the χ² test. *P*_Raw denotes the raw *P*-value, and *P*_FDR denotes the false discovery rate (FDR)-adjusted P-value. A two-tailed *P* < 0.05 was considered statistically significant

For the 10 cases of spontaneous passage, initial gastroscopy confirmed that the magnets had already migrated distal to the duodenum. These patients were managed strictly in an inpatient setting. Adhering to our institutional protocol, serial abdominal radiographs were performed every 6 h to monitor progression. The median observation duration from admission to complete spontaneous expulsion was 2 days (range: 1–3 days). To facilitate passage, conservative adjuncts such as glycerin enemas were administered when the magnets reached the distal colon or rectum. Following the retrieval of magnets from the stool and a confirmatory negative radiograph documenting complete clearance, patients were observed for an additional 24 h to ensure the absence of delayed gastrointestinal symptoms prior to discharge. Routine outpatient follow-up at one month confirmed no late complications.

### Determining the independent predictors of surgical intervention

To ascertain the independent predictors of surgical intervention, variables demonstrating statistical significance and clinical representativeness in the univariate analysis were evaluated using a multivariable logistic regression model (Table 2). The presence of diminished or absent bowel sounds was associated with the highest odds of surgery among the evaluated factors (odds ratio [OR] = 10.55; 95% confidence interval [CI]: 2.62–42.46; *P* < 0.001). Vomiting (*P* = 0.029) and abdominal pain (*P* = 0.045) were also found to be significant independent risk factors. Furthermore, an increased number of ingested magnets correlated with a higher likelihood of surgical intervention (*P* = 0.027). Conversely, a documented history of foreign body ingestion was inversely associated with the necessity of surgery (OR = 0.04; *P* = 0.003), highlighting the potential importance of prompt clinical presentation. Age at presentation did not demonstrate a significant independent association in this model (*P* = 0.689).

**Table 2:** Multivariable analysis of risk factors for surgery

Indicators	B	standard error	P value	OR	95%CI	
					lower limit	upper limit
Age at presentation (years)	-0.041	0.102	0.689	0.960	0.787	1.172
History of foreign body ingestion	-3.215	1.096	0.003	0.040	0.005	0.344
Number of foreign bodies	0.116	0.053	0.027	1.123	1.013	1.246
Abdominal pain	1.351	0.675	0.045	3.863	1.029	14.503
Vomiting	1.933	0.884	0.029	6.910	1.223	39.048
Diminished or Absent bowel sounds	2.356	0.710	<0.001	10.553	2.623	42.455

*B* regression coefficient, *OR* Odds ratio, *CI* confidence interval

Variables significant in the univariate analysis (P < 0.05) and clinically relevant were entered into the multivariable model using the forced entry method

### Imaging and diagnostic findings

All 171 patients were initially evaluated using upright anteroposterior and lateral thoracoabdominal radiographs, achieving a 100% detection rate for multiple magnetic foreign bodies (Fig. 1). Lateral views proved essential for discriminating the exact number and configuration of overlapping magnets. Plain radiography alone provided a definitive diagnosis for 157 cases (91.81%). To strictly minimize unnecessary radiation exposure and the potential need for pediatric sedation, supplementary computed tomography (CT) was not routinely performed; it was reserved exclusively for 14 complex cases (8.19%) to determine the number of magnetic foreign bodies present and detect potential complications, such as thickening of the bowel wall or localized pneumoperitoneum, which are indicative of inflammation or perforation. Crucially, although intraoperative exploration confirmed gastrointestinal perforations in 108 cases (63.16%), only 6 patients (3.51%) exhibited subdiaphragmatic free air on initial radiographs.

### Types and quantities of foreign bodies

**Fig. 2:**
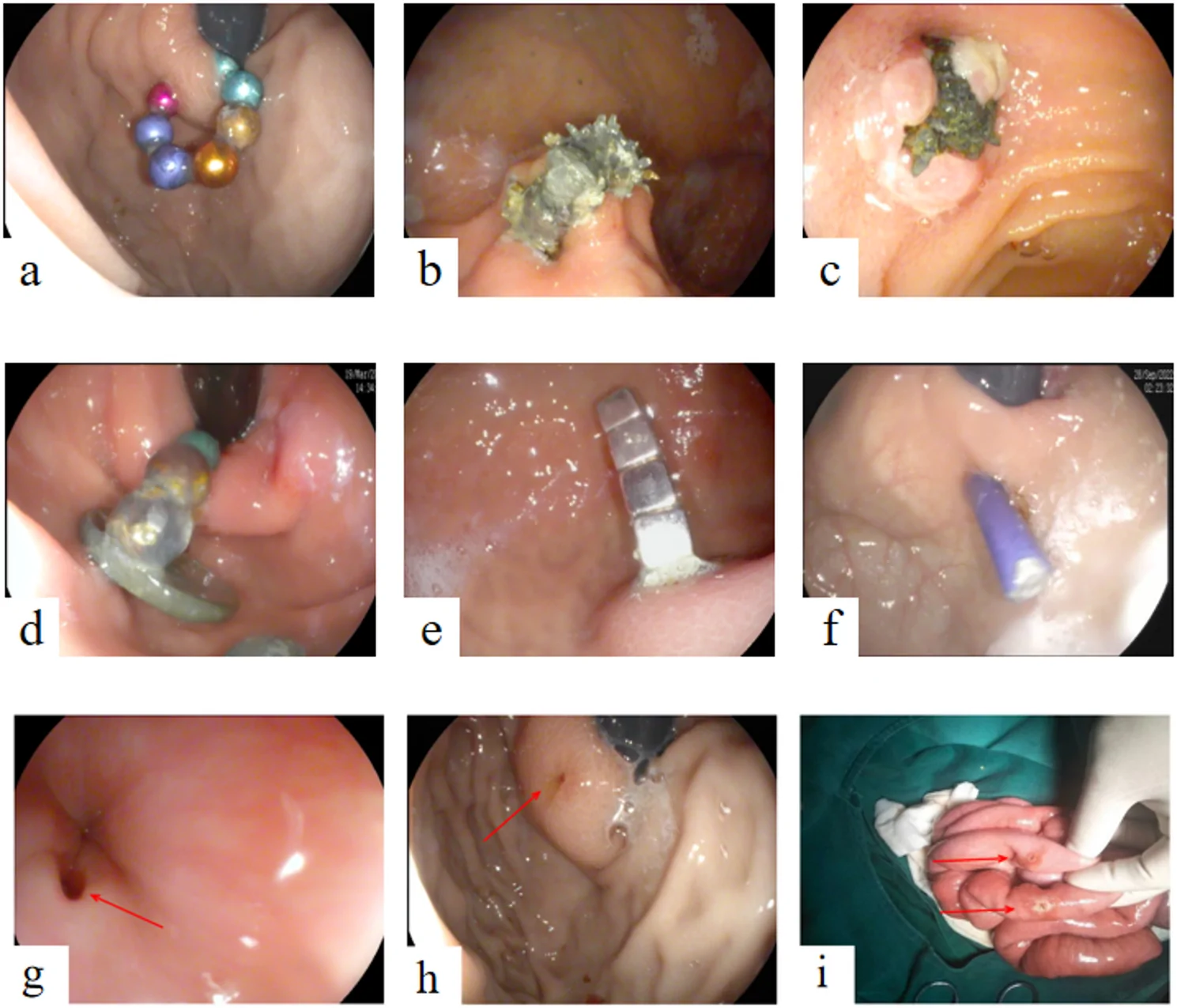
Different types of multiple magnetic foreign bodies in the digestive tract and gastrointestinal perforation. **a**, A string of magnetic beads circumferentially penetrates the cardiac incisure; **b**, Dark brown magnetic foreign bodies in the horizontal part of the duodenum, accompanied by adhesion to surrounding tissues and granulation tissue hyperplasia; **c**, Magnetic buckyballs in the small intestine; **d**, Mixed foreign bodies (Buckyballs, iron balls, and coins) at the fundus of the stomach near the cardia; **e**, Square magnetic beads at the greater curvature of the stomach; **f**, Cylindrical magnetic rods at the fundus of the stomach. **g**, Lower esophageal perforation; **h**, Perforation at the gastric fundus near the cardiac orifice; **i** Two perforations in the small intestine. The red arrows indicate the precise sites of gastrointestinal perforations

The median number of ingested magnets was 6 (range: 2–33). Pure multiple magnetic ingestions constituted 88.89% (152 cases) of the cohort. Mixed ingestions occurred in 19 cases (11.11%), involving the concurrent ingestion of non-magnetic metals (button batteries, coins, iron balls) or non-metallic objects (plastic fragments, hair, fruit pits). Morphologically, small, high-powered spherical neodymium magnetic beads (collectively referred to as “Buckyballs” in this study, regardless of their specific commercial brand) dominated the cohort (84.21%, 144 cases), alongside rarer cylindrical, oval, and square variants (Fig. 2). Notably, while five patients presented with repeated ingestions, including one diagnosed with trichophagia (pica), no patients in this cohort possessed a documented history of intentional self-harm or psychiatric disorders.

### Intraoperative and endoscopic findings

Intraoperative and endoscopic evaluations confirmed gastrointestinal perforations in 108 cases (63.16%), predominantly localizing to the small intestine or presenting as complex gastroenteric fistulas (Fig. 2). Beyond direct transmural necrosis, severe adhesive bowel disease emerged as the most frequent secondary pathology (87 cases, 50.88%). Further exploration identified a spectrum of advanced intra-abdominal catastrophes, including purulent peritonitis, mechanical bowel obstruction, mesenteric perforation, and intestinal intussusception. Detailed topographies regarding the specific locations of the magnets and resultant perforations are summarized in Table 3.

**Table 3:** Summary of the location and number of Buckyballs and perforations

Location of foreign bodies (n=171)	Number
Small intestine	56
Stomach-small intestine	19
Stomach	18
Small intestine- large intestine	17
Stomach-small intestine-large intestine	6
Stomach-esophagus	6
Duodenum	4
Stomach-large intestine	3
Duodenum-small intestine	2
Large intestine	2
Stomach-duodenum-small intestine	2
Not available^a^	31
Others^b^	5
Location of perforation	
Small intestine	50
Small intestine- large intestine	15
Stomach-small intestine	14
Stomach	9
Stomach-small intestine-large intestine	5
Others^c^	15
Number of perforations	
1	15
2	43
3	16
4	20
5	3
6	4
7	2
8	3
≥10	2
Number of multiple magnetic foreign bodies	
2	15
3	28
4	20
5	16
6	9
7	11
8	9
9	8
10≤N＜20^d^	41
20≤N＜30	9
30≤N	5

^a^ Indicates foreign body was located in the lower gastrointestinal tract and gastroscopy showed no obvious abnormalities / without gastroscopy, discharge the foreign body spontaneously, and the location was not clear

^b^ Summarizes cases with fewer sites of Buckyball (magnetic bead) retention

^c^ Summarizes cases with fewer sites of perforation

^d^ (N): Represents the number of multiple magnetic foreign bodies

### Management and outcomes of gastrointestinal perforations

Among the 108 perforation cases, 6 (5.56%) were successfully managed non-operatively following endoscopic extraction. In these specific cases, perforations were definitively confirmed via direct endoscopic visualization of the transmural defect or fistulous tract immediately following magnet retrieval. To ensure patient safety and standardize care, strict clinical criteria were applied: a “micro-defect” was defined as a focal transmural breach (estimated < 3 mm) without active leakage of gastrointestinal contents, while a “chronic fistula” was characterized by a well-contained fibrous tract completely walled off by adjacent omentum or bowel loops. Surgical intervention was deferred to minimize pediatric morbidity, as these patients met all criteria for conservative management: they maintained hemodynamic stability, lacked clinical or radiographic signs of diffuse peritonitis, and the defects were endoscopically confirmed to be anatomically contained. Therefore, they were managed medically with strict bowel rest, nasogastric decompression, broad-spectrum antimicrobial therapy, and distal nasojejunal enteral feeding. Complete mucosal healing was confirmed via follow-up endoscopy at one month.

The remaining 102 cases (94.44%) mandated surgical intervention. Necrotic margins were debrided for primary repair, while extensive necrosis or severe contamination necessitated segmental resection and anastomosis in 28 cases (27.45%). Four critical cases (3.92%) presenting with multifocal perforations and massive peritoneal contamination required temporary ileostomies. The subsequent 4-month recovery period represented the requisite physiological interval for resolving intra-abdominal inflammation and optimizing nutritional status prior to successful second-stage ostomy reversal.

**Fig. 3:**
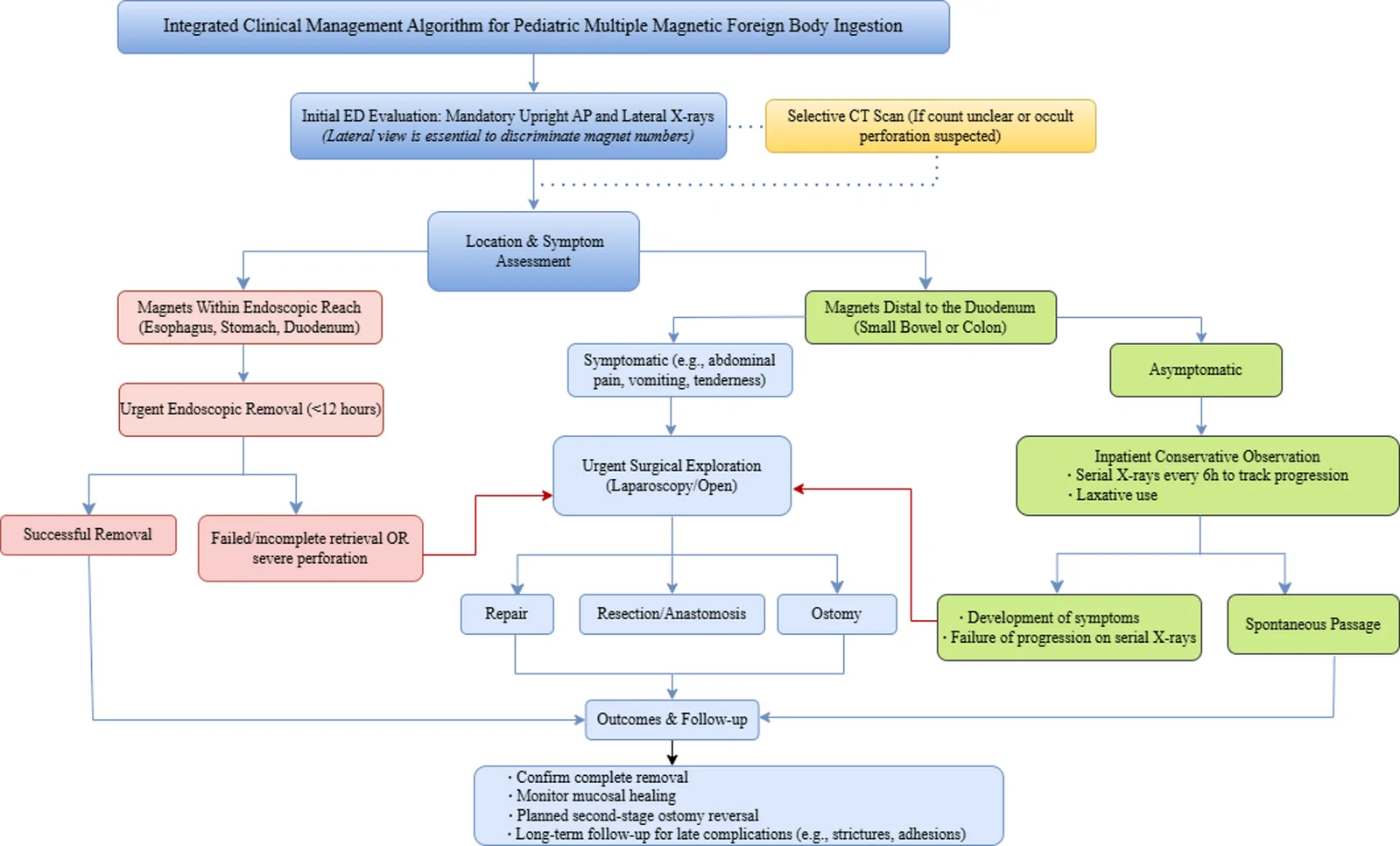
Integrated clinical management algorithm for pediatric multiple magnetic foreign body ingestion. The flowchart delineates the triage protocols based on anatomic location, symptomatology, and serial radiographic monitoring

Postoperatively, 12 patients developed complications, comprising 11 cases of adhesive small bowel obstruction (ASBO) and one case of ASBO complicated by acute ischemic intestinal necrosis. All 12 patients required and fully recovered following secondary reoperations. No hemorrhagic complications or mortalities occurred in this cohort. The overarching triage and treatment paradigms are delineated in our proposed clinical algorithm (Fig. 3).

## Discussion

This study delineates the clinical trajectory of pediatric multiple magnetic foreign body ingestions to optimize surgical triage. We demonstrate a 70.76% surgical intervention rate, identifying abdominal pain and vomiting as definitive clinical indicators of severe transmural injury, whereas witnessed ingestion facilitates timely, minimally invasive retrieval.

Beyond standard epidemiological demographics (predominantly preschool males) [10, 11], magnetic foreign body pathogenesis is governed by unique biomechanics. Magnetic attractive force is inversely proportional to the fourth power of the distance [12]; halving this distance amplifies the force sixteen-fold. Within the confined pediatric abdominal cavity, separated magnets rapidly approximate across adjacent bowel loops [5]. This sustained compression induces transmural ischemia, pressure necrosis, and subsequent fistulization or perforation, directly accounting for the 63.16% gastrointestinal perforation rate observed in our cohort, primarily involving the small bowel [13–16].

A critical diagnostic pitfall identified is the profound unreliability of plain radiography for detecting magnetic foreign body-induced perforations. Despite a 63.16% perforation rate (108 cases), only 3.51% exhibited subdiaphragmatic free air preoperatively. This discrepancy, consistent with prior reports [14, 17], stems from the magnets tightly sealing the necrotic defect, thereby precluding gross pneumoperitoneum and enteric spillage. Consequently, the absence of free air must not delay urgent surgical exploration in symptomatic patients. Furthermore, magnetic resonance imaging (MRI) remains absolutely contraindicated due to the risk of catastrophic secondary ferromagnetic tearing [18].

The clinical latency between ingestion and irreversible bowel injury severely confounds early diagnosis. Nearly half of our cohort presented without a definitive ingestion history [19], exacerbated by developmental communication barriers or deliberate concealment [20, 21]. Unwitnessed ingestions consistently correlated with advanced clinical signs—localized tenderness or absent bowel sounds—reflecting a prolonged ingestion-to-intervention interval. Aligning with Sola et al. [15] and Zhang et al. [13], our data confirm that the onset of abdominal symptoms is the most reliable harbinger of perforation, dictating an immediate shift to surgical intervention.

Therapeutic algorithms must therefore be stratified by anatomic location and symptomatology, strictly adhering to ESPGHAN [4] and NASPGHAN [5] directives. Esophagogastric magnetic foreign bodies mandate urgent endoscopic retrieval (< 12 h) to preclude distal migration. Conversely, asymptomatic patients with post-pyloric magnetic foreign bodies warrant inpatient conservative management, requiring strict monitoring with serial abdominal radiographs every 6 h [22]. Furthermore, for magnets that have progressed beyond the duodenum, balloon-assisted enteroscopy (e.g., single- or double-balloon) may serve as a valuable, minimally invasive alternative to continued observation or surgery. This approach is particularly beneficial when performed within 24 h of ingestion, a critical time window before the risk of fistula formation substantially increases [23, 24]. However, such interventions should be carefully considered based on the individual clinical scenario. It is most appropriate for asymptomatic patients with suitable anatomical size, provided there is absolutely no clinical or radiographic suspicion of bowel obstruction or perforation. Additionally, the procedure requires the institution to possess specialized equipment and advanced endoscopic expertise. While current pediatric reports on this technique remain relatively limited, and it cannot replace emergent surgical exploration when complications arise, balloon-assisted enteroscopy holds significant clinical promise in bridging the management gap for select asymptomatic cases [23, 24]. Aligning with international guidelines, this conservative approach must be immediately abandoned for surgical exploration if serial imaging demonstrates radiographic stagnation (failure of progression) or if abdominal symptoms emerge [5, 22, 25]. Given the multifocal nature of magnetic foreign body injuries, surgical intervention necessitates a comprehensive full-bowel run to identify occult perforations [26], followed by primary repair or segmental resection based on tissue viability.

Limitations of this single-center retrospective study include inherent referral and recall biases. Specifically, as a tertiary referral center, selection bias likely skews our cohort toward more complex and severe cases, potentially overestimating the overall surgical intervention and perforation rates. Furthermore, the lack of definitive ingestion timelines in unwitnessed cases introduces significant diagnostic delay. This delay not only obscures the true natural history of transmural necrosis—precluding the calculation of a precise “safe observation window” before perforation occurs—but also artificially amplifies the correlation between unwitnessed ingestions and surgical necessity. Future multicenter prospective registries are essential to mitigate these biases, standardize the observation timeframe for asymptomatic distal magnetic foreign bodies, and refine the clinical thresholds for early surgical intervention.

## Conclusions

In tertiary care cohorts, pediatric multiple magnetic foreign body ingestion frequently presents with occult transmural necrosis, demanding aggressive, algorithm-driven triage. Clinicians must recognize that the absence of radiographic pneumoperitoneum does not preclude perforation, as magnetic approximation effectively seals necrotic defects. Management must be strictly stratified by anatomy and symptomatology: urgent endoscopic retrieval is mandatory for esophagogastric magnetic foreign bodies, while asymptomatic post-pyloric ingestions require rigid serial radiographic surveillance. Crucially, the onset of abdominal symptoms or failure of progression dictates immediate surgical exploration. Ultimately, mitigating this severe surgical burden necessitates stringent legislative regulation of high-powered magnets alongside targeted caregiver education.

## Supplementary Information

Supplementary Material 1.

## Data Availability

The data that support the findings of this study are available on request from the corresponding author.

## Abbreviations

CT: Computed tomography
ASBO: Adhesive small bowel obstruction
