# Supplementary material for: Algorithm-driven management of pediatric multiple magnetic foreign bodies: a single-center experience of 171 cases
Source: BMC Pediatr. 2026 Jul 8;26:953. doi: 10.1186/s12887-026-07268-6 (PMC13640167; doi:10.1186/s12887-026-07268-6)
Supplement: Supplementary file 1 [file 12887_2026_7268_MOESM1_ESM.docx]

**Supplementary Fig. 1 Incidence of alimentary tract magnet injuries over time.**

This line graph depicts the annual number of cases with alimentary tract magnet injuries from 2019 to 2025, showing a peak in 2021 (40 cases) and a subsequent decline, with the lowest incidence in 2019 and 2025 (11 cases each).
